# Discovery of carbon-based strongest and hardest amorphous material

**DOI:** 10.1093/nsr/nwab140

**Published:** 2021-08-05

**Authors:** Shuangshuang Zhang, Zihe Li, Kun Luo, Julong He, Yufei Gao, Alexander V Soldatov, Vicente Benavides, Kaiyuan Shi, Anmin Nie, Bin Zhang, Wentao Hu, Mengdong Ma, Yong Liu, Bin Wen, Guoying Gao, Bing Liu, Yang Zhang, Yu Shu, Dongli Yu, Xiang-Feng Zhou, Zhisheng Zhao, Bo Xu, Lei Su, Guoqiang Yang, Olga P Chernogorova, Yongjun Tian

**Affiliations:** Center for High Pressure Science (CHiPS), State Key Laboratory of Metastable Materials Science and Technology, Yanshan University, Qinhuangdao 066004, China; Center for High Pressure Science (CHiPS), State Key Laboratory of Metastable Materials Science and Technology, Yanshan University, Qinhuangdao 066004, China; Center for High Pressure Science (CHiPS), State Key Laboratory of Metastable Materials Science and Technology, Yanshan University, Qinhuangdao 066004, China; Key Laboratory for Microstructural Material Physics of Hebei Province, School of Science, Yanshan University, Qinhuangdao 066004, China; Center for High Pressure Science (CHiPS), State Key Laboratory of Metastable Materials Science and Technology, Yanshan University, Qinhuangdao 066004, China; Center for High Pressure Science (CHiPS), State Key Laboratory of Metastable Materials Science and Technology, Yanshan University, Qinhuangdao 066004, China; Key Laboratory for Microstructural Material Physics of Hebei Province, School of Science, Yanshan University, Qinhuangdao 066004, China; Center for High Pressure Science (CHiPS), State Key Laboratory of Metastable Materials Science and Technology, Yanshan University, Qinhuangdao 066004, China; Department of Engineering Sciences and Mathematics, Luleå University of Technology, Luleå SE-97187, Sweden; Department of Physics, Harvard University, Cambridge, MA 02138, USA; Center for High Pressure Science and Technology Advanced Research, Shanghai 201203, China; Department of Engineering Sciences and Mathematics, Luleå University of Technology, Luleå SE-97187, Sweden; Department of Materials Science, Saarland University, Saarbrücken D-66123, Germany; Key Laboratory of Photochemistry, Institute of Chemistry, University of Chinese Academy of Sciences, Beijing 100190, China; Center for High Pressure Science (CHiPS), State Key Laboratory of Metastable Materials Science and Technology, Yanshan University, Qinhuangdao 066004, China; Center for High Pressure Science (CHiPS), State Key Laboratory of Metastable Materials Science and Technology, Yanshan University, Qinhuangdao 066004, China; Center for High Pressure Science (CHiPS), State Key Laboratory of Metastable Materials Science and Technology, Yanshan University, Qinhuangdao 066004, China; Center for High Pressure Science (CHiPS), State Key Laboratory of Metastable Materials Science and Technology, Yanshan University, Qinhuangdao 066004, China; Key Laboratory for Microstructural Material Physics of Hebei Province, School of Science, Yanshan University, Qinhuangdao 066004, China; Center for High Pressure Science (CHiPS), State Key Laboratory of Metastable Materials Science and Technology, Yanshan University, Qinhuangdao 066004, China; Center for High Pressure Science (CHiPS), State Key Laboratory of Metastable Materials Science and Technology, Yanshan University, Qinhuangdao 066004, China; Center for High Pressure Science (CHiPS), State Key Laboratory of Metastable Materials Science and Technology, Yanshan University, Qinhuangdao 066004, China; Center for High Pressure Science (CHiPS), State Key Laboratory of Metastable Materials Science and Technology, Yanshan University, Qinhuangdao 066004, China; Key Laboratory for Microstructural Material Physics of Hebei Province, School of Science, Yanshan University, Qinhuangdao 066004, China; Center for High Pressure Science (CHiPS), State Key Laboratory of Metastable Materials Science and Technology, Yanshan University, Qinhuangdao 066004, China; Center for High Pressure Science (CHiPS), State Key Laboratory of Metastable Materials Science and Technology, Yanshan University, Qinhuangdao 066004, China; Center for High Pressure Science (CHiPS), State Key Laboratory of Metastable Materials Science and Technology, Yanshan University, Qinhuangdao 066004, China; Center for High Pressure Science (CHiPS), State Key Laboratory of Metastable Materials Science and Technology, Yanshan University, Qinhuangdao 066004, China; Center for High Pressure Science (CHiPS), State Key Laboratory of Metastable Materials Science and Technology, Yanshan University, Qinhuangdao 066004, China; Key Laboratory of Photochemistry, Institute of Chemistry, University of Chinese Academy of Sciences, Beijing 100190, China; Key Laboratory of Photochemistry, Institute of Chemistry, University of Chinese Academy of Sciences, Beijing 100190, China; Baikov Institute of Metallurgy and Materials Science, Russian Academy of Sciences, Moscow 119334, Russia; Center for High Pressure Science (CHiPS), State Key Laboratory of Metastable Materials Science and Technology, Yanshan University, Qinhuangdao 066004, China

**Keywords:** amorphous carbon, ultrahard, ultrastrong, semiconductor, phase transition

## Abstract

Carbon is one of the most fascinating elements due to its structurally diverse allotropic forms stemming from its bonding varieties (*sp*, *sp*^2^ and *sp*^3^). Exploring new forms of carbon has been the eternal theme of scientific research. Herein, we report on amorphous (AM) carbon materials with a high fraction of *sp*^3^ bonding recovered from compression of fullerene C_60_ under high pressure and high temperature, previously unexplored. Analysis of photoluminescence and absorption spectra demonstrates that they are semiconducting with a bandgap range of 1.5–2.2 eV, comparable to that of widely used AM silicon. Comprehensive mechanical tests demonstrate that synthesized AM-III carbon is the hardest and strongest AM material known to date, and can scratch diamond crystal and approach its strength. The produced AM carbon materials combine outstanding mechanical and electronic properties, and may potentially be used in photovoltaic applications that require ultrahigh strength and wear resistance.

## Teaser

The currently synthesized semiconducting amorphous carbon form is the hardest and strongest amorphous material known to date, and has a bandgap comparable to that of amorphous silicon.

## Introduction

Contrary to the crystalline state of solid matter, which is characterized by periodicity in the spatial organization of the constituting atoms, the amorphous (AM) state exhibits no long-range order in the atomic arrangement although certain well-defined structural motifs may be present over a few interatomic distances, giving rise to a degree of short- to medium-range order. The length scale over which such localized ordering occurs determines the physical properties for such systems. Another example is orientational disorder of molecules perfectly positionally arranged in a crystal. In both cases a common definition of the structure of these systems is disorder (spatial and/or orientational), also termed a ‘glassy’ state. Importantly, disordered systems exhibit many properties superior to their crystalline counterparts, which make them better candidates for technological applications. Bulk metallic glasses (BMG) have physical properties combining the advantages of common metals and glasses—strength several times higher than corresponding crystalline metals, good ductility and corrosion resistance [1–3]; hydrogenated AM silicon (a-Si : H) films exhibiting an optical absorption edge at ∼1.7 eV have been the most popular photovoltaic semiconductors used in solar cells [4], and the a-Si : H/crystalline silicon (c-Si) heterojunction-based solar cell has increased efficiency steadily to a current record value of 24.7% [5], to name just a few examples. However, theoretical modeling of the AM state is prohibitively difficult, and thus, exploring new AM states of matter and their nature is both rewarding and, at the same time, a very challenging scientific task of contemporary materials science.

AM carbon exhibits a rich variety of physical properties determined by the (*sp-sp*^2^-*sp*^3^) bonding character and structural motif of the constituting atoms. Graphite-like *sp*^2^ carbon, for example, is conductive, highly compressible and flexible due to the disordered stacking of graphene layers in clusters. On the contrary, *sp*^3^ bonding-dominated diamond-like carbon (DLC) films prepared by different deposition techniques from a large variety of carbon-carrying precursors exhibit high hardness, chemical inertness and tunable optical bandgaps and, therefore, are widely used as protective coatings [6–8]. However, very large intrinsic stresses of up to several GPa in DLC films may result in the delamination of thick films from the substrates, and thereby limit the application of DLC coatings [9,10].

AM carbon can be alternatively synthesized by compression of *sp*^2^ carbon precursors, typically fullerenes and glassy carbon (GC) [11–19]. Although C_60_ molecules sustain pressure up to 20–25 GPa at ambient temperature [20], the buckyballs get easily broken at ∼5 GPa and elevated temperatures (∼800°C) to form a disordered nano-clustered graphene-based hard phase with >90% elastic recovery after deformation [21,22]. Likewise, disordered carbon materials with different *sp*^2^-*sp*^3^ carbons ratios exhibiting a remarkable combination of lightness, high strength and elasticity together with high hardness and electro-conductivity can be recovered after compressing GC at pressures of 10–25 GPa and high temperatures of ≤1200°C [11]. With a further increase of pressure the GC transforms into a metastable, *sp*^3^-rich, ultra-incompressible AM carbon [12–14]. Importantly, the synthesis of a carbon allotrope capable of scratching diamond by exposure of fullerene C_60_ to 13 GPa, 1227–1477°C, with subsequent quenching to ambient conditions, has been reported [17], although properties of this phase and interpretation of its structure remain a subject of unresolved controversy. Even though great effort has already been put into exploration of the p,T phase diagram of C_60_, a pressure range above 20 GPa has yet to be established. As synthesis pressure strongly affects the microstructure and bonding in carbon phases produced from C_60_, we may envisage the emergence of new AM carbon polymorphs as a result of crystal-to-AM and/or AM-to-AM phase transitions triggered in the pressure range of the structural integrity of C_60_ [23,24].

Here, we present a systematic study of the behavior of C_60_ fullerene at the previously unexplored pressure of 25 GPa and different temperatures. AM carbon materials, namely AM-I, AM-II and AM-III, were synthesized and characterized by complimentary techniques: X-ray diffraction (XRD), Raman spectroscopy, high-resolution transmission electron microscopy (HRTEM) and electron energy loss spectroscopy (EELS). Our results demonstrate that the *sp*^3^ carbon fraction in these materials gradually increases with the increase of synthesis temperature and, finally, reaches 69%–94%. Different hardness-measurement methods, including Knoop (*H*_K_), Vickers (*H*_V_) and nanoindentation hardness (*H*_N_), together with the uniaxial compressive strength test, were employed in order to ensure the reliability of the obtained results and demonstrate that the synthesized AM-III *bulk* material is the hardest and strongest AM material known to date. In addition, unlike insulator diamond, these AM carbon materials are semiconducting with relatively narrow bandgaps (1.5–2.2 eV) and have the potential to be used in a new class of photoelectric applications.

## RESULTS AND DISCUSSION

### Structural characterization

**Figure 1.**
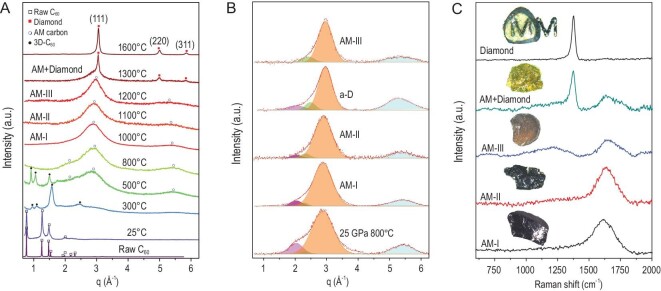
XRD patterns and Raman spectra of synthetic carbon materials collected at ambient condition. (A) XRD patterns indicating phase transition path along C_60_→3D-C_60_→AM carbon→Diamond. AM-I, AM-II and AM-III have one main diffraction peak at structure factor (q) of ∼3.0 A^–1^ as well as another weak peak around 5.3 A^–1^, which are clearly different from previously discovered low-density AM carbon materials from compressing C_60_ at relatively low pressures of 6.5–13 GPa [17]. (B) Peak fitting of the XRD patterns of the AM carbon materials and a-D from compressing GC [12]. The magenta, green, orange and light-blue peaks are at q = ∼2.0 Å^–1^, ∼2.4 Å^–1^, ∼3.0 Å^–1^ and ∼5.3 Å^–1^, respectively. The peak at q = ∼2.0 Å^–1^ in a-D [12], AM-I, AM-II and the AM carbon recovered from compressing C_60_ at 25 GPa and 800°C, originates from the interlayer diffraction signal of residual graphite-like nanoclusters in the structure. This peak disappears in AM-III, demonstrating the formation of a completely different short-range ordered structure. (C) UV Raman spectra of AM-I, AM-II, AM-III, AM + Diamond composite and diamond. The insets are the optical photographs of recovered samples, displaying that AM-III is yellow-transparent and distinct from the black AM-I and AM-II.

Figure 1A and B show XRD patterns of the materials recovered after treatment of C_60_ at 25 GPa and various synthesis temperatures. The following sequence of phase transitions was observed: first, C_60_ transforms into the known 3D polymer [17] at elevated temperature (new sharp diffraction peaks appear), then buckyball destruction/structure amorphization begins at ∼500°C (very broad new peaks appear and the intensity of the polymer peaks decreases) and completes above 800°C. The materials recovered from 1000°C, 1100°C and 1200°C, termed AM-I, AM-II and AM-III, respectively, are characterized by a dominant broad diffraction peak centered near q = ∼3.0 Å^–1^, fairly close to the position of (111) reflection of diamond (q = 3.05 Å^–1^), and a weaker peak at q = ∼5.3 Å^–1^ (Fig. 1A and Supplementary Fig. 1 in the online supplementary file), which represent an entirely new class of AM carbon material distinctly different from the previously reported low-density AM carbon materials synthesized at lower pressures and temperatures (13 GPa, 1227–1477°C) [17]. Recently, Shi and Tanaka revealed that the first sharp diffraction peak (FSDP) in tetrahedral covalent AM materials such as Si, Ge and C comes from the characteristic density waves of a single tetrahedral unit, and the integrated intensity of the FSDP is directly proportional to the fraction of locally favored tetrahedral structure or a measure of the tetrahedrality [25]. Notably, previously discovered AM carbon materials have another graphite-like diffraction peak near q = ∼2.0 Å^–1^ indicating large interlayer spacing and lower density [18] (see also the results of our test experiment conducted at similar conditions, as described in ref. [17], Supplementary Fig. 2). When the synthesis temperature increases from 1000°C to 1200°C, the AM peaks become slightly narrower and shift from ∼2.88 to ∼3.00 Å^–1^, indicating further density increase. Also, the material's color changes from opaque black to transparent yellow (insets in Fig. 1C). As the synthesis temperature exceeds 1300°C, the narrow diffraction peaks corresponding to (111), (220) and (311) reflections of diamond appear near 3.05, 4.98 and 5.84 Å^–1^, respectively, indicating the formation of nanocrystalline diamond (nano-diamond) coexisting with the remaining AM phase.

The bonding difference in the AM carbon materials is reflected in their Raman spectra (Fig. 1C and Supplementary Fig. 3). The AM-I and AM-II exhibit a broad Raman peak around 1600 cm^–1^ with full width at half peak maximum (FWHM) of ∼200 cm^–1^, corresponding to the G-band characteristic of *sp*^2^ carbons. Appearance of the G-band peak testifies to a relatively high fraction of *sp*^2^ bonded carbon atoms [26]. Indeed, accounting for a very low Raman cross section for *sp*^2^ carbon at UV laser excitation, the high intensity of the G-band in the spectra of AM-I and AM-II clearly indicates the presence of relatively high *sp*^2^ carbon atoms in these AM materials. Importantly, both position and the FWHM of the G-band peak indicate that the Raman scatterers’ (clusters’) size in these materials must be <2 nm [27]. On the contrary, the background-subtracted Raman spectrum of AM-III reveals several new features. First, a band located at the low wavenumbers of 900–1300 cm^–1^ (termed ‘T-band’ [27]) is a characteristic signature of *sp*^3^ carbon and thus indicates their high concentration in the AM-III. Second, an evident shoulder (rising peak) on the high frequency side of the G-band (at 1740 cm^–1^) may be attributed to clustering (cross-linking via *sp*^3^ bonds) of remaining aromatic rings formed of *sp*^2^ carbon and, finally, the peak appearing at ∼1930 cm^–1^ likely originates from short linear chains (Supplementary Fig. 3B). After completion of the AM-diamond transformation above 1600°C, the fingerprint peak of crystalline diamond at ∼1330 cm^–1^ appears in the spectra of transparent diamond samples (see the topmost inset in Fig. 1C).

**Figure 2.**
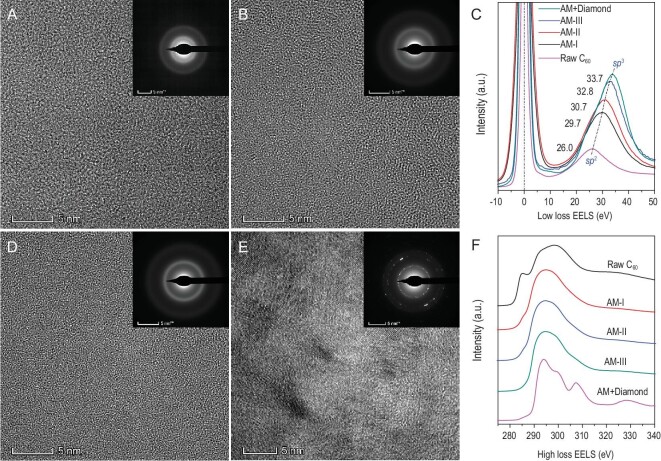
Microstructure and bonding of synthetic carbon materials. (A, B and D) HRTEM images of AM-I, AM-II and AM-III, respectively, showing their uniform disorder characteristics and gradually decreased AM fragment sizes. The insets: the corresponding SAED patterns exhibit two diffuse rings near 2.1 Å and 1.2 Å. (E) HRTEM image of AM + Diamond composite. The results of the HRTEM and SAED patterns indicate the formation of nanocrystalline diamond. (C) Low-loss EELS data show that the position of the plasmon peak is shifted from 26.0 eV to 33.7 eV, indicating the increase of *sp*^3^ content in the samples. (F) High-loss EELS data show that the contribution of the *sp*^2^ carbon in the spectra represented by the 1s-π^*^ (285 eV) transition gradually decreases with the increase of synthesis temperature (top to bottom).

In order to confirm the microstructure and bonding nature of the AM carbon materials suggested by Raman, HRTEM, selected area electron diffraction (SAED) and EELS were performed. The SAED patterns display two diffuse rings near 2.1 Å and 1.2 Å in all three AM carbon materials (Fig. 2), which is consistent with the XRD results. For comparison, the composite sample recovered from 1300°C shows, in addition, the ‘spotty’ diffraction rings indicating the formation of nanocrystalline diamond. The main feature of the low-loss EELS data in Fig. 2C is a gradual shift of the plasmon peak from its position in pristine C_60_ (26.0 eV) to higher energies in AM-I, AM-II and AM-III (29.7, 30.7 and 32.8 eV, respectively) that demonstrates an increase of *sp*^3^ fraction in the AM carbon materials. The plasmon peak position in AM-III is higher than that in the ‘AM diamond’ (a-D) produced by quenching GC from high p,T (31.8 eV) and implies lower *sp*^3^ content and density in the latter [12]. According to the plasmon peak position in the low-loss EELS spectra (Supplementary Fig. 4A), the *sp*^3^ fraction in AM-I, AM-II and AM-III was estimated to be 69 ± 4%, 77 ± 2% and 94 ± 1%, respectively, similar to the method described previously [28]. Density of AM-I, AM-II and AM-III was directly measured at ∼2.80 ± 0.17, ∼2.96 ± 0.08, and ∼3.30 ± 0.08 g/cm^3^, respectively, thus demonstrating AM-III is the densest AM carbon approaching diamond. The *sp*^3^ fraction value was also independently determined based on the density of AM carbon materials using the calibration plot of *sp*^3^ fraction vs. density [29], as shown in Supplementary Fig. 4B, which is similar to above results estimated from plasmon peak position. In addition, the peak at 285 eV in carbon K-edge (high-loss) EELS signaling the *sp*^2^ fraction in the material gradually decreases when going from AM-I to AM-III (Fig. 2F). The linear EELS scans with high spatial resolution in randomly selected sample regions demonstrate the bonding homogeneity at least on a 1 nm scale in these AM carbon materials (Supplementary Fig. 5). The subtle microstructure differences between the AM carbon materials are further revealed by HRTEM images that exhibit a characteristic ‘worm-like’ contrast manifesting structural disorder (Fig. 2A, B and D). The dimensions of these very fine structural fragments gradually decrease with the synthesis temperature increase, reaching a statistically averaged size of ∼12 Å, 8 Å and only 4 Å in AM-I, AM-II and AM-III, respectively. That clearly distinguishes these disordered carbon materials from those containing a substantially lower fraction of *sp*^3^-bonded atoms obtained from GC at similar p,T conditions [11], underscoring the importance of the precursor material selection in high p,T synthesis.

### Mechanical properties

**Figure 3.**
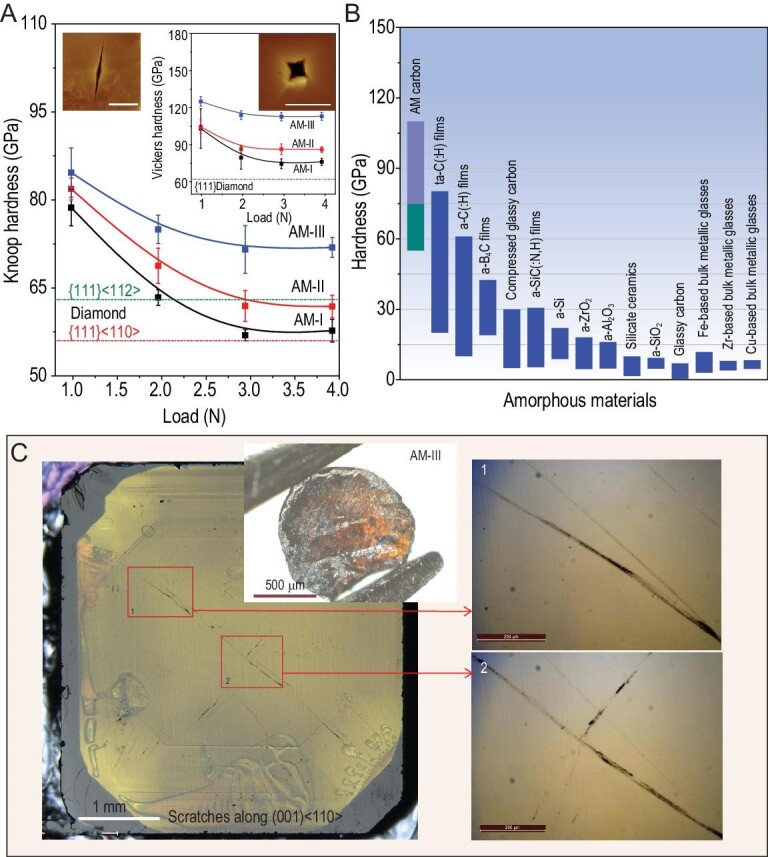
Hardness of AM carbon materials, compared with other known AM materials, and scratches on diamond (001) face indented by AM-III. (A) *H*_K_ as a function of applied loads. Left inset: AFM image of Knoop indentation of AM-III after unloading from 3.92 N. Right inset: *H*_V_ of AM carbon materials as a function of applied load and AFM image of Vickers indentation of AM-III sample after unloading from 2.94 N. The scale bars in the indentation images are 10 μm. Error bars of hardness indicate s.d. (*n* = 5). The dashed lines indicate *H*_V_ and *H*_K_ of (111) plane of natural diamond crystal. (B) Hardness of different AM materials [1,4,8,11,33]. Green and violet columns indicate *H*_K_ and *H*_V_ of AM carbon materials, respectively. Considering the hardness of film materials are characterized by nanoindentation hardness (*H*_N_), the *H*_N_ of AM carbon materials was also measured, and AM-III has a high *H*_N_ of 103 GPa, exceeding that (80.2 GPa) of ta-C films [8]. (C) Scratches on the (001) face of diamond by using an AM-III sample displayed in the inset as an indenter (left image), indicating the ultrahard nature of AM-III. The zoomed-in right images correspond to the areas marked by red rectangles in the left image, displaying the scratches in more detail.

The hardness values, i.e. *H*_K_, *H*_V_ and *H*_N_, of the AM carbon materials were estimated by three independent measurement methods. The results as well as detailed indentation images are presented in Fig. 3 and Supplementary Figs 6 and 7. Among the synthesized materials, AM-III has the highest hardness of *H*_K _= 72 ± 1.7 GPa and *H*_V_ = 113 ± 3.3 GPa, whereas the AM-I and AM-II have *H*_K_ of 58 ± 1.9 and 62 ± 1.9 GPa, respectively. In comparison, the *H*_V_ and *H*_K_ values of the (111) plane of natural single crystalline diamond are 62 and 56 GPa [30,31], respectively (Fig. 3A and Supplementary Fig. 8), thus the hardness of synthesized AM carbon materials can rival that of diamond. Careful analysis of Vickers indentation morphologies of AM-III shows that the raised ‘pile-up’ was formed due to flow of the displaced material up around the indenter, indicating the plastic character of the deformation during loading (Supplementary Fig. 9C). With the applied load increase up to 3.92 N, the radial and lateral cracks as well as the peeling zone can be observed around the resultant indentations (Supplementary Fig. 9A and B), implying the occurrence of the plastic-to-brittle transition in the material [32]. Moreover, the *H*_N_ and Young's modulus (*E*) have also been determined based on the load-displacement curves using the Oliver and Pharr model [33] (Supplementary Fig. 7). The estimated *E* of AM-I, AM-II and AM-III are 747 ± 66, 912 ± 89 and 1113 ± 110 GPa, respectively. The obtained *H*_N_ for them are 76 ± 3.4, 90 ± 7.9 and 103 ± 2.3 GPa, respectively, which are comparable to their Vickers hardness. Notably, the *H*_N_ of AM-III exceeds the record of 80.2 GPa held until now by tetrahedral AM carbon (ta-C) films [8]. Such extreme hardness allows the AM-III sample to scratch the (001) face of synthetic diamond crystal with an *H*_V_ of 103 GPa (Fig. 3C and Supplementary Fig. 8A). Possessing hardness comparable to that of single crystalline diamond, AM-III becomes the hardest AM material known to date (Fig. 3B). More significantly, the advantage of this ultrahard AM carbon is that it has isotropic hardness comparable to diamond crystals where the hardness varies along different crystallographic directions leading to a cleavage of diamond that easily occurs along its ‘weak’ crystal planes.

**Figure 4.**
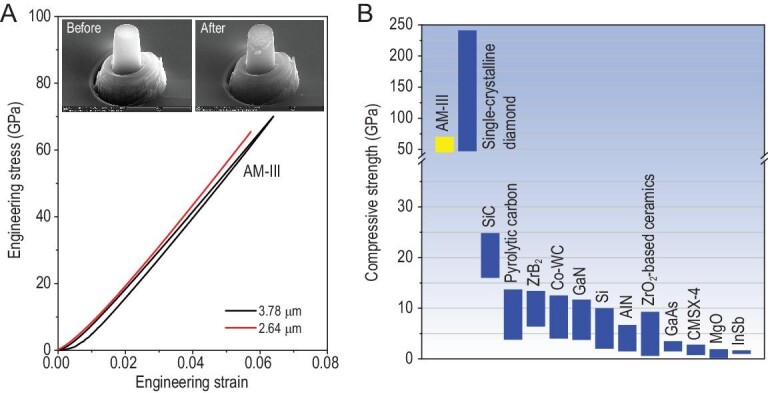
Compressive strength of AM-III compared with other known materials. (A) Engineering stress-strain curves recorded during uniaxial compressing of AM-III micropillars. The insets are the SEM images of the pillar with a diameter of 3.78 μm before and after compression. There was almost no size change, but some wrinkles produced on the upper part of the pillar are like the shear bands formed in metallic glasses [3]. (B) Comparison of compressive strength for various materials with micron size [34–36]. The results demonstrate that AM-III is the strongest AM material known to date.

The superior mechanical properties of AM-III have been further demonstrated by *in-situ* uniaxial compression/decompression test (Supplementary Fig. 10). It was found that a micropillar made out of the AM-III with a top diameter of 0.88 μm has compressive strength of at least 40 GPa, and could be fully elastically recovered without fracture after decompression at ambient conditions. Subsequent measurement of a micropillar with a larger top diameter (3.78 μm) demonstrated its ability to withstand compressive stress as high as ∼70 GPa without fracture although in this case a closer examination of the decompressed pillar revealed some wrinkles produced in its upper part (insets in Fig. 4A), very similar to the shear bands formed in metallic glasses during deformation [3]. Another AM-III micropillar with a diameter of 2.64 μm was broken at a stress load of 65 GPa before reaching its strength limit. Thus the measured compressive strength of AM-III lies in between that of <100>- and <111>-oriented diamond micropillars exhibiting the compressive strength of ∼50 GPa and ∼120 GPa, respectively [34]. Theoretically, the maximum compressive strength of materials can only be obtained when the load is strictly perpendicular to the top surface of the pillar, a condition that is very difficult to achieve. As a result, the value of the ideal compressive strength of the AM carbon pillars should, in fact, be higher than the value we determined in our experiment. Consequently, our measurements demonstrate that the AM-III is comparable in strength to diamond and superior to the other known high-strength materials (Fig. 4B) [34–36].

It is important to ascertain what may be the reason(s) for the observed AM carbon materials with an *sp*^3^ carbon fraction below 100%, in particular AM-I with only 69% *sp*^3^ carbon fraction, exhibiting hardness and strength comparable to that of crystalline diamond. Indeed, it is well known, the *sp*, *sp*^2^ and *sp*^3^ covalent bonds in elemental carbon are all extremely strong. For example, the intrinsic strength of graphene (pure *sp*^2^ carbon) reaches a value as high as 130 GPa [37] thus exceeding the ultimate tensile strength of diamond <111> direction (95 GPa [38]) comprised of *sp*^3^ carbon atoms. The fundamental reason for the softness of graphite is weak van der Waals interaction between graphene layers. However, high pressure induces partial *sp*^2^-to-*sp*^3^ transformation leading to interlinking/locking-in the graphene layers by the tetrahedral *sp*^3^ bonds and profound increase of hardness and strength of the resulting high-pressure phase that is able to abrade the diamond anvils [39]. The *sp*^2^-*sp*^3^ carbon system with only 22% *sp*^3^ fraction experimentally obtained at ambient conditions by quenching from high-pressure compressed GC has a high hardness of 26 GPa [11], whereas the three-dimensional (3D) C_60_ polymer comprised of covalently linked (via *sp*^3^ bonds) fullerene molecules with ∼40% *sp*^3^ carbon content possesses a superhigh hardness of 45 GPa [40]. Moreover, a number of superhard/ultrahard *sp*^2^-*sp*^3^ crystalline carbon forms were recently predicted theoretically. For example, the carbons designated as P-1-16b, P-1-16e and P-1-16c with ∼50% *sp*^3^ carbons are predicted to have an ultrahigh hardness of 71.3–72.4 GPa [41]. A series of *sp*^2^-*sp*^3^ 3D carbon nanotube polymers were also predicted to have superhigh hardness, such as the 3D (8,0) nanotube polymer with 43.5% *sp*^3^ carbon predicted to have a hardness of 54.5 GPa [42,43]. A class of diamond-graphene (diaphite) nanocomposites constructed from covalently connected *sp*^3^-diamond and *sp*^2^-graphite structural units are predicted to have increased hardness and improved fracture toughness [44,45]. All the above-mentioned experimental and theoretical results demonstrate that ultrahigh hardness and strength comparable to crystalline diamond can be achieved in *sp*^2^-*sp*^3^ carbon systems at *sp*^3^ concentrations below 100%. The AM carbon materials synthesized in this work have higher *sp*^3^ content than compressed GC [11] and 3D-C_60_ polymers [40], and thus we may anticipate higher hardness and strength in our systems. More importantly, it is not just a fraction of *sp*^3^ carbon atoms that matters in this case but the structural motif. We argue that our *sp*^2^-*sp*^3^ carbon systems represent a particular short-range order that is a ‘blend’ of remaining *sp*^2^ carbon-based units (fused aromatic rings, short chains) covalently interlinked with clusters of tetragonally coordinated *sp*^3^ carbons. Such a ‘blend’, represented on the HRTEM images (Fig. 2A, B and D) by worm-like structural fragments, must combine the nearly intrinsic graphene-type strength/hardness of the *sp*^2^ units with the diamond-like strength/hardness of the clusters formed by tetragonally coordinated *sp*^3^ carbon. That may explain why even AM-I, with a relatively low *sp*^3^ fraction, is competitive in hardness and strength with crystalline diamond. In the development of substantially smaller structural fragments (fused rings opening, interlinking the structural units via short chains) along with a significant increase of *sp*^3^ fraction in AM-III, a new short-range order must emerge and further manifest in a profound increase of hardness and strength, and an alteration of the optical properties of the system.

### Optical properties

**Figure 5.**
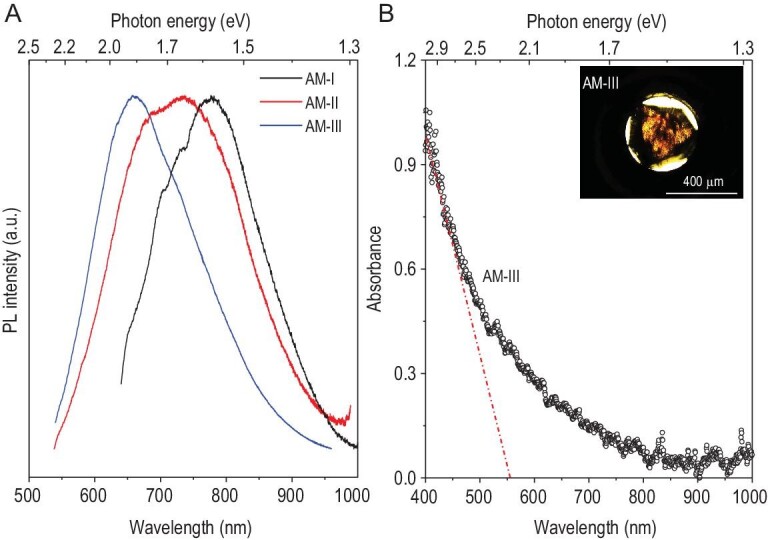
Optical properties and bandgaps of AM carbon materials. (A) PL spectra measured at ambient condition. The AM-I spectrum is excited by 633 nm laser, the AM-II and AM-III spectra are excited by 532 nm laser. The bandgaps of AM carbon materials estimated from PL spectra are between 1.5 and 2.2 eV, illustrating their semiconducting nature. (B) Absorption spectrum of AM-III. The absorption edge of AM-III is at ∼570 nm, corresponding to an optical bandgap value of 2.15 eV. The inset shows an optical microscope view of a piece of transparent AM-III placed inside the hole of a gasket that is mounted inside the diamond-anvil cell (DAC).

The AM carbon materials under investigation also display unusual optical properties. All the materials exhibit strong photoluminescence (PL) in the range of 550–950 nm when excited by a 532 or 633 nm laser (Fig. 5A). The PL maxima correspond to photon energies of 1.59 ± 0.1, 1.74 ± 0.2 and 1.87 ± 0.1 eV, in AM-I, AM-II and AM-III, respectively. This difference is directly related to the higher content of *sp*^3^ carbon-based material possessing larger bandgaps in the samples. In view of the yellow-transparent nature of AM-III, its visible light absorption spectrum was measured in transmission utilizing a diamond anvil cell (DAC). The inset of Fig. 5B shows the view in transmitted light through a sample piece mounted in a gasket hole inside the DAC. The result indicates that the optical absorption edge of AM-III is located at ∼570 nm, which corresponds to a bandgap of 2.15 eV, consistent with the PL results. Therefore, these AM carbon materials are a class of semiconductors with bandgaps less than diamond (5.5 eV) and close to the AM silicon (a-Si : H) films (∼1.7 eV) wildly used in technology nowadays. These preferable optical bandgaps mean there is the potential to use these AM carbon materials as optimal semiconductors for novel photoelectric applications.

### Comparison of various types of AM carbon materials

**Figure 6.**
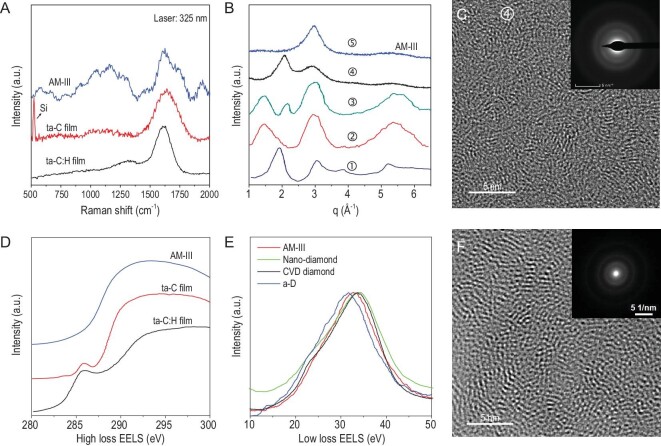
Comparison of AM-III with other AM carbon materials. (A) Raman spectra show AM-III has an obvious T-band around 900–1300 cm^–1^ compared to ta-C(:H) films [8,27]. (B) XRD patterns of various AM carbon materials recovered from compressing C_60_ at p,T conditions: ① 8 GPa, 1200°C [17]; ② 12.5 GPa, 500°C [17]; ③ 13.5 GPa, 1000°C [17]; ④ 15 GPa, 800°C (our data); ⑤ 25 GPa, 1200°C (AM-III, our data). (C and F) HRTEM images and SAED patterns of the material synthesized at p,T condition ④ and compressed GC [11], respectively. (D) High-loss EELS of AM-III and ta-C(:H) films [46,47]. (E) Low-loss EELS demonstrating the plasmon peak position in AM-III, a-D [12], CVD diamond [48] and nano-diamond [49].

It is important to define the position of the materials we produced on the current landscape of other technologically important (hard) AM carbon-based materials. The data reported/published to date can be divided into two categories according to the preparation method: thin films prepared by various deposition routes [8,27,46,47] and the materials synthesized at high-pressure and high-temperature using different precursors such as fullerene [17,18] and GC [11,12]. Further, we mainly focus on the most distinct material—AM-III (Fig. 6). Comparing the microstructure and bonding of the discovered AM-III with ta-C(:H) films [8,27,46,47] through the corresponding UV Raman and EELS (Fig. 6A, D and E), one can see a much stronger Raman T-band around 900–1300 cm^–1^ characteristic of *sp*^3^ carbon and a negligible EELS intensity in the AM-III against the peak near 285 eV representing residual *sp*^2^ carbon in ta-C(:H) films [46,47]. Importantly, in the films, the residual *sp*^2^ carbon presents as orientationally disordered nano-sized graphene clusters whereas no graphene-based structural units survive 25 GPa synthesis pressure in the AM-III we report here. The evident structural difference results in a significant performance difference between these materials. For example, the AM-III has a high *H*_N_ of 103 GPa, which is comparable to the hardest crystal plane of diamond, and higher than that (80.2 GPa) of the reported ‘hardest’ ta-C film [8].

In the second category, the hard AM carbon materials were produced at high p,T from fullerene and GC precursors with synthesis pressures up to 15 GPa [17] and 50 GPa [12], respectively. The XRD patterns in Fig. 6B exhibit a clear difference between AM-III and various AM carbon materials synthesized previously by compressing C_60_ at relatively low synthesis pressures (up to 15 GPa) [17]—the graphite-like diffraction peaks near q = ∼1.5–2.0 Å^–1^ still appear in the XRD patterns, indicating the presence of large interlayer spacings and, consequently, relatively low densities. These highly disordered *sp*^2^ carbon-based systems exhibit graphene-nanocluster-derived short-range order that is preserved at the synthesis pressure used in earlier experiments [11,17], which is evidenced in both Raman and HRTEM data (Fig. 6A and C). In order to further reveal the characteristics of this type of AM carbon material, we made a special effort to perform synthesis at p,T conditions (15 GPa, 550–1200°C, see Supplementary Fig. 2) similar to those used in ref. [17]. The Vickers hardness of the material we synthesized at 15 GPa, 800°C (see its HRTEM in Fig. 6C) was found to be 68 GPa, i.e. lower than that of newly synthesized AM carbon materials (Fig. 3A), therefore (i) testifying to the presence of an entirely different type of short-range order and composition (*sp*^2^/*sp*^3^ ratio) in the system and (ii) demonstrating that fullerene compression at a level of 25 GPa is an essential requirement to facilitate both the alteration of the short-range order (crushing the residual nano-graphene clusters) and *sp*^2^ to *sp*^3^ transformation/formation of the tetragonal AM carbon matrix. On the contrary, using GC comprised of relatively large, irregular and curved multilayer graphene sheets as the precursor demonstrated that one must go to much higher pressures than 25 GPa in order to create *sp*^3^ carbon-based material, as graphene nanoclusters formed by crushing the curved graphene sheets in GC survive at this synthesis pressure and exhibit super-elastic properties when quenched to ambient conditions (see its HRTEM in Fig. 6F) [11]. Indeed, laser heating to ∼1527°C at 40–50 GPa allowed the production of a *sp*^3^-rich system, the so-called ‘quenchable a-D’ [12]. The XRD pattern of a-D reveals the signature of a residual peak at ∼2 Å^–1^ corresponding to a graphite-like interlayer distance, and low EELS data indicate higher residual *sp*^2^ carbon content in a-D compared to that in AM-III (Fig. 1B). The comparison between AM produced from GC, and the materials synthesized in this work, demonstrates the ultimate importance of the precursor material in the high p,T synthesis. Indeed, using a highly symmetrical intrinsically nanostructured C_60_ molecule (only ∼7 Å in diameter) as a precursor provides uniform breaking and conversion of the bonds along with amorphization of the structure under 25 GPa, 1000–1200°C compared to GC, where even an increase of pressure to 50 GPa was insufficient to turn it at ∼1527°C into a uniform *sp*^3^ carbon-based structure.

Thus, the AM carbon can be divided into at least five categories according to our understanding, by summarizing all the reported and currently synthesized materials. *The first type* is all-*sp*^2^ disordered carbon materials composed of curved graphite-like or multilayer graphene fragments with variable sizes and microstructures (e.g. five-, six- or seven-membered rings), such as carbon black, GC and other AM carbon materials formed from high-temperature carbonization of organic compounds. *The second type* is mainly composed of curved multilayer graphene fragments with variable sizes and microstructures, and a small amount of *sp*^3^ bonds formed between the layers of multilayer graphene fragments, such as compressed GC [11] and a-C(:H) films formed by deposition [6,7]. For this kind of AM carbon material, an obvious graphite-like diffraction of q = ∼1.5–2.0 Å^–1^ can be observed (Fig. 6B, sample ①). Compared with *the first type* of all-*sp*^2^ AM carbon, this type of AM carbon material has significantly improved mechanical properties such as high hardness/strength, but also has good conductivity due to the *sp*^2^ bonding dominant. *The third type* is composed of an *sp*^3^-dominant dense disordered component and disordered nano-multilayer graphene fragments, such as ta-C(:H) films [8,27,46,47] and currently synthesized AM-I and AM-II, as well as the AM carbon materials recovered from compressing C_60_ at 15 GPa and 800–1000°C. For this kind of AM carbon material, the diffraction peak from multilayer graphene in the structure at q = ∼2.0 Å^–1^ still exists, but becomes weak. At the same time, the intensity of the diffraction peak at q = ∼3.0 Å^–1^ from the diamond-like tetrahedral structure gradually increases with the transformation and decrease of the multilayer graphene component in the microstructure. This type of AM carbon is a semiconducting material with superhigh hardness and strength. *The fourth type* is composed of an *sp*^3^-dominant dense disordered structure, such as AM-III, currently synthesized. This type of AM carbon material has no diffraction peak from the multilayer graphene interlayer (q = ∼1.5–2.0 Å^–1^) and only has a broad diffraction peak centered at ∼3.0 Å^–1^, which is close to the position of (111) reflection of diamond. *The fifth type* is an ideal AM carbon with complete *sp*^3^-bonded carbon atoms.

Going forward we must underscore that contrary to crystalline materials, where using just one technique, XRD, for example, is sufficient for distinguishing different structural states, a complimentary characterization of the AM carbon materials is mandatory as it allows for clear identification of different states of disordered matter. Only by using complimentary characterization comprised of XRD, Raman, HRTEM and EELS could we not only distinguish the newly synthesized AM carbon materials from all other AM carbon materials reported to date, but also reveal subtle differences between these structural forms of carbon. For example, although the difference between AM-III and AM-I/AM-II is evident, the latter materials are hard to distinguish when we just look at their Raman spectra (Fig. 1C and Supplementary Fig. 3). On the contrary, the EELS data indicate the difference in *sp*^3^ fraction between all the AM carbon materials (Fig. 2C and F, and Supplementary Fig. 4), and the HRTEM demonstrates the homogeneous contrast but distinct difference in the size of the structural worm-like fragments in the AM carbon materials (Fig. 2A, B and D). We infer that evolution from the AM-I to AM-II state likely goes via relaxation of the structure around crushed buckyballs triggered by a temperature increase at 25 GPa—fusion of the remaining aromatic rings built of *sp*^2^ carbons, further carbon conversion from the *sp*^2^ to *sp*^3^ state and bridging the fused rings and clusters of tetragonally-coordinated *sp*^3^ atoms. A more profound change in the short-range order occurs in AM-III leading to the aromatic rings opening and short chains forming (evidenced by the appearance of a new Raman peak at 1940 cm^–1^, Supplementary Fig. 3B), accompanied by interlinking of the structural elements via *sp*^3^ carbon, the fraction of which substantially increases on this step. Consequently, these structural differences result in the different performances of the AM carbon materials, particularly the mechanical and optical properties as discussed in detail above.

The above analysis demonstrates that the discovered AM-III is indeed a new AM carbon material never detected or reported before. The distinct short-range order, microstructure and composition provide a unique combination of semiconducting and superior mechanical properties (with hardness and strength at the level of natural/synthetic diamond).

## CONCLUSION

In summary, by extending synthesis pressure to 25 GPa the AM carbon materials were created from C_60_ precursor. Higher synthesis pressure seizes the growth of graphene clusters after buckyballs collapse leading to high enrichment of the synthesized disordered phases with *sp*^3^-bonded carbon, thus concluding the search for a bulk material based on a tetragonally arranged *sp*^3^ carbon network and finally complimenting and expanding the technological value of existing 2D systems—ta-C and DLC films. Consequently, the materials exhibit outstanding mechanical properties—comparable to crystalline diamond—and the hardness and strength of AM-III surpass any known AM material. Thermal stability of AM-III in-air is comparable to that of diamond crystals [30] (Supplementary Fig. 11). Remarkably, these AM carbon materials are all semiconductors with bandgaps in the range of 1.5–2.2 eV. The emergence of this type of ultrahard, ultrastrong, semiconducting AM carbon material offers an excellent candidate for the most demanding of practical applications and calls out for further experimental and theoretical exploration of AM carbon allotropes.

## METHODS

### Sample synthesis

Samples with diameters of ∼1 mm and heights of 1.2–1.7 mm were recovered after compressing C_60_ fullerene (99.99%, Alfa Aesar) at a pressure of 25 GPa and high temperatures. The standard COMPRES 8/3 assembly consisting of an 8-mm-spinel (MgAl_2_O_3_) octahedron with a Re heater and a LaCrO_3_ thermal insulator was used for high-pressure (p ∼25 GPa) and high-temperature (T ∼2300°C) experiments in a large-volume multi-anvil system at Yanshan University, identical to the one described elsewhere [30]. Pressure loading/unloading rates were 2 GPa/hour, heating rate was 20°C/min, maintained for 2 hours and finally quenched by turning off the electric power supply.

### X-ray diffraction and Raman spectroscopy

XRD was performed on a Bruker D8 Discover diffractometer with Cu *Kα* radiation source. Both Raman scattering and PL measurements were carried out on a Horiba Jobin–Yvon LabRAM HR-Evolution Raman microscope at ambient conditions. The Raman spectra were excited by laser radiation of 325 nm, and the PL spectra were excited by 532 or 633 nm laser. In all experiments the laser beams were focused to a spot size of ∼1 μm.

### HRTEM and EELS measurements

Samples for HRTEM were prepared by a Ga focused ion beam (FIB) (Scios, FEI) milling with an accelerating voltage of 30 kV. HRTEM, SAED and EELS measurements were carried out at Themis Z TEM, using an accelerating voltage of 300 kV. The EELS spectra were collected in the TEM mode at a random region of ∼200 nm. The EELS line scans were conducted in scanning transmission electron microscopy (STEM) mode with an energy resolution of 0.6 eV and spatial resolution of ∼1 nm.

### Hardness and elastic modulus measurement

*H*_K_ and *H*_V_ were measured by microhardness tester (KB 5 BVZ). *H*_N_ and Young's moduli (*E*) were measured at the peak load of 0.98 N with Berkovich diamond indenter (Keysight Nano Indenter G200). The indentations were imaged by the Atomic Force Microscope (AFM) to obtain an accurate hardness. The scratch test was conducted using AM-III as an indenter to scratch the (001) crystal face of diamond.

### Compressive strength test

The micropillars with diameters of ∼1 to 4 μm and aspect ratios of ∼1.5 to 2.5 were fabricated using a Ga ion beam in the FIB instrument (Scios, FEI). The compression measurements were conducted using a PI 87 PicoIndenter system interfaced with a Helios NanoLab DualBeam microscope and nanoindentation system (Keysight Nano Indenter G200).

### Optical absorption

The VIS/NIR absorption spectra were recorded on a UV/VIS/IR spectrometer (Avantes, AvaSpec) using a Xenon Light Source by assembling a sample piece with a thickness of ∼50 μm into a DAC with a culet size of 500 μm. The bandgaps were derived from the absorption spectra using the method described elsewhere [50].

### Thermal stability measurement

Differential scanning calorimetry (DSC) and thermogravimetric analysis (TGA) using NETZSCH STA 449F5 were measured in the temperature range of 25–1400°C with a heating rate of 10°C/min.

## Supplementary Material

nwab140_Supplemental_File
