# Supplementary material for: Discovery of carbon-based strongest and hardest amorphous material
Source: Natl Sci Rev. 2021 Aug 5;9(1):nwab140. doi: 10.1093/nsr/nwab140 (PMC8776544; doi:10.1093/nsr/nwab140)
Supplement: nwab140_Supplemental_File [file nwab140_supplemental_file.docx]

**Supplementary Information**

**Discovery of carbon-based strongest and hardest amorphous material**

**Shuangshuang Zhang^1†^, Zihe Li^1†^, Kun Luo^1,2†^, Julong He^1†^, Yufei Gao^1,2†^, Alexander V. Soldatov^3,4,5^, Vicente Benavides^3,6^, Kaiyuan Shi^7^, Anmin Nie^1^, Bin Zhang^1^, Wentao Hu^1^, Mengdong Ma^1^, Yong Liu^2^, Bin Wen^1^, Guoying Gao^1^, Bing Liu^1^, Yang Zhang^1,2^, Yu Shu^1^, Dongli Yu^1^, Xiang-Feng Zhou^1^, Zhisheng Zhao^1^*, Bo Xu^1^, Lei Su^7^, Guoqiang Yang^7^, Olga P. Chernogorova^8^, Yongjun Tian^1^***

^1^Center for High Pressure Science (CHiPS), State Key Laboratory of Metastable Materials Science and Technology, Yanshan University, Qinhuangdao, Hebei 066004, China

^2^Hebei Key Laboratory of Microstructural Material Physics, School of Science, Yanshan University, Qinhuangdao 066004, China

^3^Department of Engineering Sciences and Mathematics, Luleå University of Technology, SE-97187 Luleå, Sweden

^4^Department of Physics, Harvard University, Cambridge, MA 02138, USA

^5^Center for High Pressure Science and Technology Advanced Research, Shanghai 201203, China

^6^Department of Materials Science, Saarland University, Campus D3.3, D-66123, Saarbrücken, Germany

^7^Key Laboratory of Photochemistry, Institute of Chemistry, University of Chinese Academy of Sciences, Chinese Academy of Sciences, Beijing, 100190, China

^8^Baikov Institute of Metallurgy and Materials Science, Moscow 119334, Russia

* Corresponding authors: [zzhao@ysu.edu.cn](mailto:zzhao@ysu.edu.cn) (Z.Z.) or [fhcl@ysu.edu.cn](mailto:fhcl@ysu.edu.cn) (Y.T.). †These authors contributed equally to this work.

**Table of Contents**

- **Figure 1.** Structure factors S(q) of the carbon materials quenched from synthesis pressure of 25 GPa and various temperatures.
- **Figure 2.** XRD patterns of carbon materials synthesized at 15 GPa and different temperatures from collapsed C_60_ fullerenes collected after subsequent samples quenching to ambient conditions.
- **Figure 3.** UV Raman spectra of the carbon materials quenched from synthesis pressure of 25 GPa and various temperatures.
- **Figure 4.** Variation of *sp*^3^ fraction vs. plasmon energy for various carbon materials (A), and variation of density vs. *sp*^3^ fraction for various carbon materials (B).
- **Figure 5.** EELS mappings of AM-I (A) and AM-III (B).
- **Figure 6.** Images of Knoop, Vickers and Berkovich indentations.
- **Figure 7.** Nanoindentation hardness (A) and Young’s moduli of AM-I, AM-II and AM-III (B).
- **Figure 8.** Vickers (A) and Knoop (B) hardness of different crystal faces of single crystalline diamond.
- **Figure 9.** Vickers indentation morphologies of AM-III after unloading from different loads (A and B) and the corresponding indentation profiles along the diagonals scanned by AFM (C).
- **Figure 10.** *In-situ* compression/decompression testing of an AM-III micron-sized pillar*.*
- **Figure 11.** Thermogravimetric analysis (TGA) (top panel) and differential scanning calorimetry (DSC) heat flow data (bottom panel) collected from AM-III in air.

**Figure 1.** Structure factors S(q) of the carbon materials quenched from synthesis pressure of 25 GPa and various temperatures. Two broad diffraction peaks are visible at positions of ~3.0 and 5.3 Å^-1^, respectively. With the synthesis temperature increase, the first and second peak shift to higher- and lower q, gradually approaching the (111) and (220) reflections position of diamond at q=3.05 and 4.98 Å^-1^, respectively. The dashed lines give an indication of the peak shifts.

**Figure 2.** XRD patterns of carbon materials synthesized at 15 GPa and different temperatures from collapsed C_60_ fullerenes collected after subsequent samples quenching to ambient conditions. With the increased synthesis temperatures, the resulting phase transition path is C_60_→3D-C_60_→ Amorphous carbon→Diamond/compressed graphite composite. At this synthesis condition, the AM carbon materials recovered from 700-1000 °C have two main diffraction peaks at around 2.0 Å^-1^ and 2.9 Å^-1^, as well as one minor peak at about 5.3 Å^-1^. In contrast, the AM carbon materials recovered from 25 GPa and 1000-1200 °C have only two diffraction peaks, at around 3.0 Å^-1^ and 5.3 Å^-1^. The diffraction peak centered around 2.0 Å^-1^ corresponds to the graphite interlayer-like distance of ~3.1 Å.

**Figure 3.** UV Raman spectra of the carbon materials quenched from synthesis pressure of 25 GPa and various temperatures. (A) The fluorescence background was not removed. Above 1300 °C, the Raman peak of diamond appeared at 1330 cm^-1^. Below that synthesis temperature, all the spectra exhibit characteristic of *sp*^2^ carbon broad G-band located at about 1600 cm^-1^. An additional broad peak appears at around 900~1300 cm^-1^ in AM-III, which is commonly known as T-band indicating the high *sp*^3^ carbons fraction in this material. (B) Decomposition of the UV Raman spectrum of the AM-III and peaks assignment. Peaks 1 to 5: UV Raman spectroscopy of hydrocarbons [1] reveals similar vibrations that can be related to different configuration of fused aromatic rings. In case of AM-III it is likely that the aromatic rings are linked to each other (fused) via *sp*^3^ carbons and randomly oriented in the material; F-Band: this vibration is related to pentagonal rings (analogous to Ag(2) mode in C_60_). F-band was observed in fullerene-like disordered carbon systems [2], like glassy carbon [3], fullerene-like amorphous carbon thin films [4, 5] and nano-clustered graphene [6]; G-Band peak position reflects a mixture of linear chains and fused rings fragments [2]; “C-clustering” peak at 1740 cm^-1^ corresponds to tiny graphene clusters residues in highly disordered *sp*^3^/*sp*^2^ carbon systems, for example, carbon nanodots [7]; peak at 1940 cm^-1^ is tentatively assigned to short linear carbon chains [2, 8].


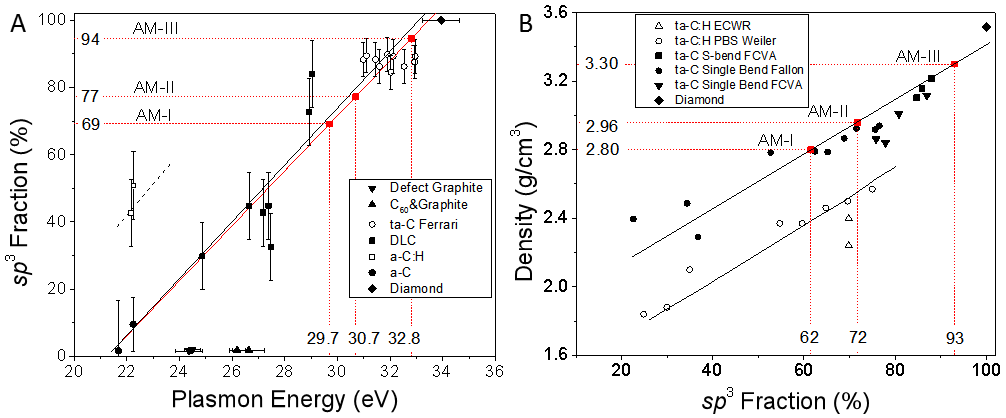


**Figure 4.** (A) Variation of *sp*^3^ fraction vs. plasmon energy for various carbon materials [11]; the red line is the result of linear re-fitting, based on the combined data after adding the Ferrari’ data [12]. (B) Variation of density vs. *sp*^3^ fraction for various carbon materials [13]. The *sp*^3^ fraction in AM-I, AM-II, and AM-III was estimated according to above linear relationship (see red dots).

**Figure 5.** EELS line scans conducted in STEM mode along ~120 nm long lines with 1 nm step and energy resolution of 0.6 eV in a randomly selected regions of the AM-I (A) and AM-III (B) samples, respectively, showing the bonding homogeneity in their microstructures.

**Figure 6.** Images of Knoop, Vickers and Berkovich indentations. (A, B and C) AFM images of Knoop indentations on surfaces of AM-I, AM-II and AM-III phases, respectively. In all cases, the applied load was 3.92 N. (D) Optical and SEM images of AM-III phase surface after indentation at a load of 0.98 N with Berkovich-type pyramid probe. (E) AFM image of a residual indentation by the Vickers probe on AM-III surface at a load of 2.94 N. The inset shows corresponding optical photograph of the indentation. (F) AFM scan of the indentation profile in e along the diagonals.

**Figure 7.** Nanoindentation hardness and Young’s moduli of AM-I, AM-II and AM-III. (A) Loading/unloading displacement curves during indentation measurement. The derived hardness (*H*_N_) at a peak load of 0.98 N are 76±3.4, 90±7.9, and 103±2.3 GPa, respectively, which are comparable to the hardness values determined by Vickers method. (B) Young’s moduli (*E*) of the AM carbon materials. By assuming Poisson’s ratio of 0.2, the estimated *E* of AM-I, AM-II and AM-III are 747±66, 912±89, 1113±110 GPa, respectively.

**Figure 8.** Vickers (*H*_V_) and Knoop (*H*_K_) hardness of different crystal faces of single crystalline diamond. (A) *H*_V_ as a function of applied load. The asymptotic *H*_V_ values of {111} and {110} faces of natural diamond are 62 and 111 GPa, respectively [14]. In this work we determined the asymptotic *H*_V_ of {001} face of synthetic diamond at 103 GPa. (B) *H*_K_ of natural diamond along different crystallographic directions. The data in the figure are from the literature [15] (I: Type Ia natural diamond, and II: Type IIa natural diamond).

**Figure 9.** Vickers indentation morphologies of AM-III after unloading from different loads (A and B) and the corresponding indentation profiles along the diagonals scanned by AFM (C). At small loads, there is no obvious indentation cracks, indicating the dominant plastic deformation. At the large loads, the radial and lateral cracks as well as peeling zones can be found around the indentations, demonstrating the plastic-to-brittle transition [16]. For all the loads, the displaced material flows up around the indenter to form a raised pile-up, indicating the occurrence of plastic flow in these cases.

**Figure 10.** *In-situ* compression/decompression testing of an AM-III micron-sized pillar. (A) *In-situ* SEM images exhibit the pillar height change during compression (①-⑤) and after decompression ⑥. The micron-sized pillar with a top diameter of 0.88 µm was shortened during compression, and the deformation was completely recovered after unloading. (B) Engineering stress-strain curve. Notably, the compression curve shows nonlinearity at the maximum load, likely due to tilting and bending of the pillar.

**Figure 11.** Thermogravimetric analysis (TGA) (top panel) and differential scanning calorimetry (DSC) heat flow data (bottom panel) collected from AM-III in air. The oxidation onset temperatures were determined at 734 °C and 687 °C, from TGA and DSC data, respectively. The thermal stability of AM-III is comparable to that of single crystalline diamond [14].

**REFERENCES**

1. Loppnow GR, Shoute L and Schmidt KJ, et al. UV Raman spectroscopy of hydrocarbons. *Phil Trans R Soc Lond A* 2004; **362**:2461–2476.

2. Ferrari AC and Robertson J. Resonant Raman spectroscopy of disordered, amorphous, and diamondlike carbon. *Phys Rev B* 2001; **64**:075414.

3. Solopova NA, Dubrovinskaia N and Dubrovinsky L. Raman spectroscopy of glassy carbon up to 60 GPa. *Appl Phys Lett* 2013; **102**:121909.

4. Wang Q, Wang C and Wang Z, et al. Fullerene nanostructure-induced excellent mechanical properties in hydrogenated amorphous carbon. *Appl Phys Lett* 2007; **91**:141902.

5. Dennison JR, and Doyle TE. An embedded ring approach to the vibrational dynamics of low-dimensional amorphous solids with applications to graphitic carbon materials. *Carbon* 1997; **35**:1465–1477.

6. Chernogorova O, Potapova I and Drozdova E, et al.Structure and physical properties of nanoclustered graphene synthesized from C_60_ fullerene under high pressure and high temperature. *Appl Phys Lett* 2014; **104**:043110.

7. Bhattacharyya S, Ehrat F and Urban P, et al. Effect of nitrogen atom positioning on the trade-off between emissive and photocatalytic properties of carbon dots. *Nat Commun* 2017; **8**:1401.

8. Buntov EA, Zatsepin AF, Guseva MB and Ponosov YuS. 2D-ordered kinked carbyne chains: DFT modeling and Raman characterization. *Carbon* 2017; **117**:271–278.

9. Zeng Z, Yang L and Zeng Q, et al. Synthesis of quenchable amorphous diamond. *Nat Commun* 2017; **8**:322.

10. Serin V, Beche E and Abidate O, et al. Proceedings of the fifth international symposium on diamond materials. *Electrochem Soc Proc* 1998; **97–32**:124126–141

11. Fallon PJ and Brown LM. Analysis of chemical-vapour-deposited diamond grain boundaries using transmission electron microscopy and parallel electron energy loss spectroscopy in a scanning transmission electron microscope. *Diam Relat Mater* 1993; **2**:1004–1011.

12. Ferrari AC, Kleinsorge B and Morrison NA, et al. Stress reduction and bond stability during thermal annealing of tetrahedral amorphous carbon. *J Appl Phys* 1999; **85**:7191–7197.

13. Libassi A, Ferrari AC and Stolojan V, et al. Density and sp^3^ content in diamond-like carbon films by x-ray reflectivity and electron energy loss spectroscopy. *MRS Proceedings* 1999; **593**: 293-298.

14. Huang Q, Yu D and Xu B, et al. Nanotwinned diamond with unprecedented hardness and stability. *Nature* 2014; **510**:250–253.

15. Brookes CA and Brookes EJ. Diamond in perspective: A review of mechanical properties of natural diamond. *Diam Relat Mater* 1993; **1**:13–17.

16. Donovan PE. Plastic flow and fracture of Pd_40_Ni_40_P_20_ metallic glass under an indentor. *J Mater Sci* 1989, **24**:523–535.
